# Can Artificial Intelligence Beat Humans in Detecting Breast Malignancy on Mammograms?

**DOI:** 10.7759/cureus.46208

**Published:** 2023-09-29

**Authors:** Mariam Malik, Saeeda Yasmin, Anish Kumar, Yumna Hassan, Yusra Rizvi, Iffat

**Affiliations:** 1 Radiology, Atomic Energy Cancer Hospital, Nuclear Medicine, Oncology and Radiotherapy Institute (NORI), Islamabad, PAK; 2 Internal Medicine, Fatima Jinnah Medical University, Lahore, PAK; 3 Internal Medicine, Ghulam Muhammad Mahar Medical College and Hospital, Sukkur, PAK; 4 Internal Medicine, Insight Hospital and Medical Center Chicago, Chicago, USA; 5 Internal Medicine, Dow University of Health Sciences, Karachi, PAK

**Keywords:** screening mammograms, screening of breast cancer, breast cancer, artificial intelligence and breast cancer, computer-aided detection

## Abstract

Background: The study was aimed at identifying how useful Computer-Aided Detection (CAD) could be in reducing false-negative reporting in mammography and early detection of breast cancer at an early stage as the best protection is early detection.

Materials and methods: This retrospective study was conducted in a tertiary care setup of Atomic Energy Cancer Hospital, Nuclear Medicine, Oncology and Radiotherapy Institute (AECH-NORI), where 33 patients with suspicious findings on mammography and subsequent biopsy-proven malignancy were included. The findings of mammography including the lesion type, breast parenchymal density, and sensitivity of CAD detection, as well as the final biopsy results, were recorded. A second group of 40 normal screening mammograms was also included who had no symptoms, had Breast Imaging-Reporting and Data System category I(BI-RADS I) mammograms, and had no pathology identified on correlative sonomammography as well.

Results: A total of 35 masses, 11 pleomorphic clusters of microcalcification, five clustered foci of macrocalcification, and nine lesions with pleomorphic clusters of microcalcification and two with pleomorphic clusters of microcalcification only were included. The CAD system was able to identify 26 masses (74%), eight lesions with pleomorphic clusters of microcalcification (72%), five foci of macrocalcification (100%), six lesions with pleomorphic clusters of microcalcification (66%), and two pleomorphic clusters of microcalcification without formed mass (100%). The overall sensitivity of the CAD system was 75.8%. CAD was able to identify 13 out of 16 masses with invasive ductal carcinoma (81.3%), eight out of nine lesions proven as invasive ductal carcinoma with ductal carcinoma in situ (DCIS) (88.9%), two out of five masses with invasive lobular carcinoma (40%), four out of four masses with invasive mammary carcinoma (100%), and zero out of one lesion identified as medullary carcinoma (0%). There was 100% detection for pleomorphic clusters of microcalcification without formed mass with CAD marking two out of two mammograms.

Conclusion: CAD performed better with combined lesions, accurately marked pleomorphic clusters of microcalcification, and identified small lesions in predominant fibrofatty parenchymal density but was not reliable in dense breast, areas of asymmetric increased density, summation artifacts, edematous breast parenchyma, and retroareolar lesions. It also performed poorly with ill-defined lesions of invasive lobular carcinoma. Human intelligence hence beats CAD for the diagnosis of breast malignancy in mammograms as per our experience.

## Introduction

Breast cancer has become the most commonly diagnosed cancer worldwide [1]. Its rising trend may be attributable to various environmental and genetic factors [2]. However, improved diagnostic techniques and awareness have led to early detection of this disease [3,4] and, subsequently, the increase in the number of diagnosed cases. The alarming rise in the number of breast cancer cases has led to advancements in diagnostic techniques, as early detection has a better prognosis than late detection [5]. It also has led healthcare systems worldwide to start screening programs [6]. These programs have undoubtedly influenced the survival rates as screen-detected cancer responded better to treatment than patients who presented with some symptoms [7]. However, this has increased the burden on already strained healthcare systems globally and has led to the introduction of artificial intelligence (AI)-based screening programs for screening purposes [8].

Image checker was the first Computer-Aided Detection (CAD) approved by the Food and Drug Administration (FDA) for diagnostic and screening programs [9]. It works like a spellchecker for images [10] and analyzes images derived from the tomosynthesis dataset [11,12] using software algorithms to mark suspicious findings linked with breast cancer [13,14]. The software uses the symbols to mark the site of abnormality including sites of calcification, masses, and a composite mark for lesions suggestive of calcification and masses in the same area [15].

CAD is able to mark clusters with three or more elements that are within 3 mm, and each element is 150 microns in size [15]. It is able to detect the presence of asymmetry in breast size [16], regions of radiating lines, suspicious masses, and microcalcification [17]. It also occasionally marks calcified arteries, benign calcifications, crossing breast tissues, ducts, and areas of parenchymal summation, well-circumscribed masses, lymph nodes, nipple retraction, and vague opacities. Similarly, the likelihood of the detection of a suspicious lesion also increases with the increased density of the lesion and spiculated margins, as well as the detection of associated asymmetry in comparison with the contralateral normal breast [18]. The CAD software compares the findings on craniocaudal (CC) and mediolateral oblique (MLO) projections as well as between the right and left breast tissue. The system verifies the findings to see if they match the criteria of the software and marks the area with a symbol in the center of the suspicious area. The algorithm does not mark low-volume foci, lead skin markers, or clips. The areas marked by CAD are reviewed by radiologists to ascertain whether these are truly of concern [18].

Studies have indicated an increased frequency of detection of breast cancer with CAD when compared with double reading [19]. A review of the literature has also shown a favorable performance of CAD in the detection of abnormalities, but it cannot be used alone for the detection or diagnosis [20] of breast cancer. However, in our experience of reporting mammograms, we have seen that CAD has frequently missed lesions in increased breast parenchymal density and, in certain subsets, types of lesions.

We have taken a dataset of a certain number of patients to see the sensitivity of this software in the detection of malignant masses and neoplastic calcifications to see if CAD software has better results than human intelligence. This would particularly help us identify if this software can be used to assist less experienced radiologists in detecting breast pathology.

## Materials and methods

We conducted a retrospective consecutive cross-sectional study in our tertiary care cancer hospital between July 2022 (the time we started using the CAD software as a supplementary tool for our mammogram imaging) and November 2022 (the time of the completion of our sample size necessary to ensure the study’s statistical power and generalizability) for patients who underwent bilateral digital mammography. The mammograms were performed on the Selenia model of Hologic, and two standard views, namely, CC and MLO projections, were available for reporting for every patient. Consecutive sampling was used to avoid selection bias.

Patient selection for patients with breast masses

We included 45 consecutive female patients who had undergone screening and diagnostic studies with subsequent trucut biopsy in our hospital for the breast lump proven on histopathology as a carcinoma and/or pleomorphic cluster of microcalcification that was subsequently proven to be mitotic on histopathology results. There were no males or transgender included as our patient population comprised only females. The reporting of mammograms was done by senior radiologists with more than five years of mammography reporting experience. All the patients had a correlative ultrasound performed by the same radiologists. Finalized reports of imaging as well as the imaging were stored in and retrieved from the picture archiving and communication system (PACS) as well as the Hospital/Health Management Information System (HMIS). Histopathologies were also retrieved from the HMIS record. The mean age of these patients was 51.5 years (range: 35-65 years). All other patients in whom a suspicious lesion was seen on mammography but subsequent histopathology results were not available for those who did have ultrasound imaging after mammogram were excluded.

Patient selection for patients with normal mammograms

Forty consecutive screening mammograms were selected for females who had no symptoms and subsequently had normal mammography results with no abnormality seen on correlative sonomammography. All these mammograms were evaluated by CAD to determine false-positive markings, and these normal mammograms with CAD markings were considered false-positive. The mean age of these patients was 48.5 years (range: 35-58 years).

Analysis of mammograms with the CAD system

Full-field digital mammography (FFDM) generates two-dimensional (2D) images for viewing on a review workstation (processed images) or processing by other software (raw images). The raw images are sent from the server software to PACS and then to Image Checker CAD 10.0. CAD scrutinizes the images producing results as a “.xml” file and sends the output data back to the server software. The server software henceforth creates the results in the form of a DICOM Mammography CAD SR object, which contains the CAD marks. The CAD results are displayed alongside the FFDM images. CAD marks densities and masses, areas of architectural distortion, calcification, and mass with calcification.

For each patient in our study, the processed images were reviewed with correlative ultrasound. A biopsy was done for the identified masses, and a histopathology report was followed. The processed images in retrospect were then compared with CAD marked images. The performance of CAD was identified by the number of correctly marked pathologies and analyzed using a chi-square analysis. For normal mammograms, the numbers of false-positive markings by CAD were identified for masses and calcifications.

Data analysis

Results were entered in Statistical Package for Social Sciences (SPSS) version 20 (IBM SPSS Statistics, Armonk, NY, USA) and analyzed according to frequencies and percentages for both the patients with breast masses and those with normal mammograms.

## Results

CAD performance: Breast carcinoma

Among the 45 patients with breast pathology identified on mammography, 33 had histopathologically proven carcinoma. In these 33 patients, there were a total of 35 masses, 11 pleomorphic clusters of microcalcification, five clustered foci of macrocalcification, and nine lesions with pleomorphic clusters of microcalcification and two with pleomorphic clusters of microcalcification only. The CAD system was able to identify 26 masses (74%), eight lesions with pleomorphic clusters of microcalcification (88.9%), five foci of macrocalcification (100%), and two pleomorphic clusters of microcalcification without formed mass (100%). The overall sensitivity of the CAD system, hence, was 75.8% (that is, correctly identifying 47 pathologies out of a total of 62). The findings are detailed in tabulated form in Table 1.

**Table 1:** Total number and percentage of types of pathology identified correctly by CAD CAD: Computer-Aided Detection

Pathology	Total number present	Total number identified by CAD	Percentage identified by CAD (%)
Masses	35	26	74
Total number of pleomorphic clusters of microcalcification (including those identified with and without formed mass)	11	10	91
Mass with pleomorphic cluster of microcalcification	9	8	88.9
Pleomorphic cluster of microcalcification without formed mass	2	2	100
Clustered foci of macrocalcification	5	5	100

The histopathological analysis of 35 masses included invasive ductal carcinoma in 16 masses, invasive ductal carcinoma with ductal carcinoma in situ (DCIS) in nine lesions, invasive lobular carcinoma in five masses, medullary carcinoma in one lesion, and invasive mammary carcinoma in four lesions, while pleomorphic clusters of microcalcification only were histopathologically proven as DCIS. CAD was able to identify 13 out of 16 masses with invasive ductal carcinoma (81.3%), eight out of nine lesions proven as invasive ductal carcinoma with DCIS (88.9%), two out of five masses with invasive lobular carcinoma (40%), four out of four masses with invasive mammary carcinoma (100%), and zero out of one lesion identified as medullary carcinoma (0%). There was 100% detection for pleomorphic clusters of microcalcification without formed mass with CAD marking two out of two mammograms. The findings are detailed in tabulated form in Table 2.

**Table 2:** Percentage of tumor types correctly identified by CAD CAD: Computer-Aided Detection, DCIS: ductal carcinoma in situ

Pathology	Total number	Number identified by CAD	Percentage identified by CAD (%)
Invasive ductal carcinoma	16	13	81.3
Invasive ductal carcinoma with DCIS	9	8	88.9
Invasive lobular carcinoma	5	2	40
Medullary carcinoma	1	0	0
Invasive mammary carcinoma	4	4	100
DCIS only	2	2	100

**Figure 1:**
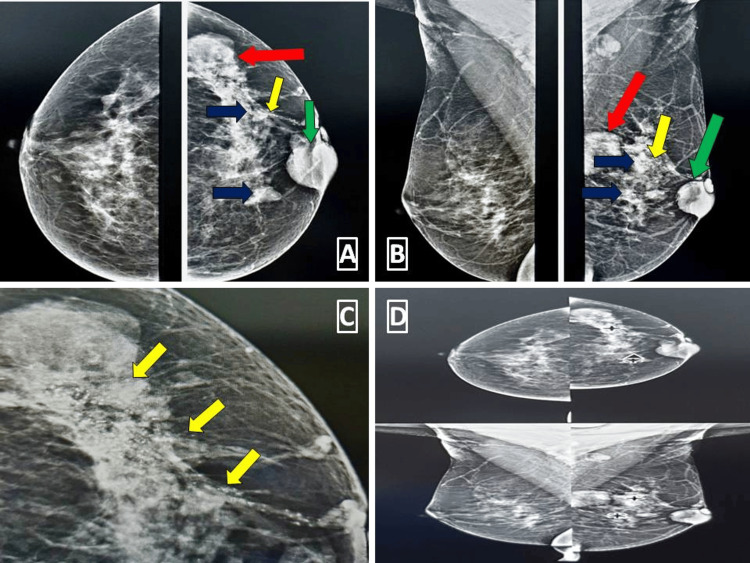
Mammogram images of type C breast parenchyma (dense breast) obscuring fine details The craniocaudal and mediolateral projections of the left breast (Figure 1A and Figure 1B) demonstrate parenchymal heterogeneity with scattered areas of architectural distortion. A well-circumscribed lesion is seen in the upper outer quadrant as indicated by red arrows. A retroareolar lesion is present in the left breast indicated by green arrows. Additionally, two smaller radiodense lesions are also faintly visible in the upper outer and lower inner quadrants indicated by blue arrows. There are also pleomorphic clusters of microcalcification in a ductal distribution in the upper inner quadrant, more clearly evident in the magnified Figure 1C indicated by the yellow arrows. CAD has identified the smaller lesions and pleomorphic clusters of microcalcification as seen in Figure 1D but failed to mark the retroareolar lesion and the lesion in the upper outer quadrant. The right breast is normal (BI-RADS 1). CAD: Computer-Aided Detection, BI-RADS 1: Breast Imaging-Reporting and Data System category I

The lesions proven to be invasive ductal carcinoma on histopathology were identified by lesion type, sensitivity of CAD detection, and density of breast parenchyma. Lesion types were as follows: 16 masses on mammograms without microcalcification, eight masses on mammograms with pleomorphic clusters of microcalcification (mixed pathology), and one mammogram with pleomorphic clusters of microcalcification without any lesion. It was observed that CAD performed poorly in dense breasts and more often missed lesions masked by the overlapping parenchyma, especially in lesions smaller than 2 cm in size. CAD also was not reliable in inflammatory carcinomas with breast edema findings and was also unable to mark retroareolar lesions, which measured up to 1 cm in maximum transverse dimension in one of our patient’s mammography (Figure 1A-1D). For invasive ductal carcinoma presenting as a lesion only on a mammogram, the CAD sensitivity was 81.3% versus 88.9% for invasive ductal carcinoma with pleomorphic clusters of microcalcification, the results favoring a better sensitivity of CAD for mixed pathology. For pleomorphic clusters of microcalcification without formed lesions proven as DCIS via stereotactic biopsy results, CAD showed a sensitivity of 100%. CAD hence showed an overall good performance in the detection of pleomorphic clusters of microcalcification with or without a mass.

**Figure 2:**
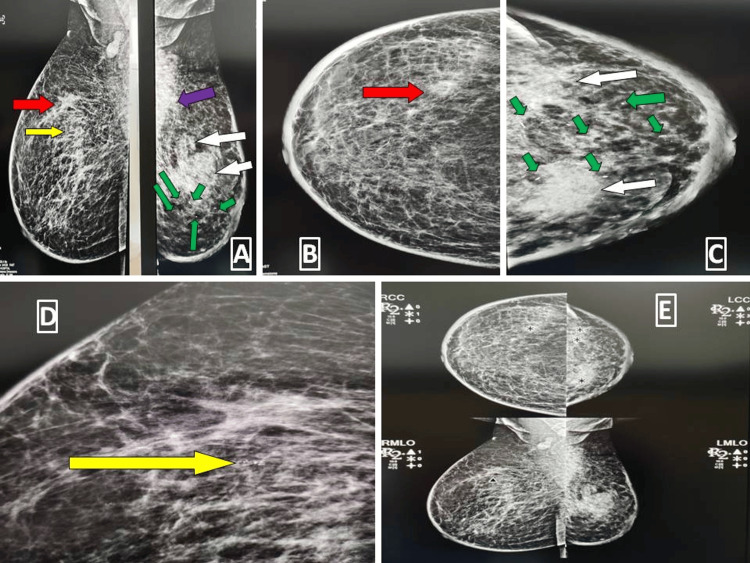
Patient with bilateral inflammatory invasive lobular carcinoma Mammogram shows ACR category C breast parenchyma bilaterally. There is an ill-defined lesion in the upper outer quadrant of the right breast as marked by red arrows in Figure 2A and Figure 2B. A small pleomorphic cluster of microcalcification is visible only on mediolateral oblique projection (indicated by the yellow arrow in Figure 2A) and better identified in magnified Figure 2D (yellow arrow). There are two ill-defined lesions in the upper outer and upper inner quadrants of the left breast (identified by white arrows). An ill-defined lesion in the right axilla is indicated on the MLO view in Figure 2A by the purple arrow. Scattered foci of macrocalcification are also present in the left breast (indicated by green arrows) seen in Figure 2A and Figure 2C. Figure 2E shows that CAD has marked the lesions correctly in craniocaudal projection but missed the lesions completely in MLO view. A pleomorphic cluster of microcalcification is marked in MLO view. The discrete scattered foci of macrocalcification in the left breast are missed by CAD. ACR: American College of Radiology, MLO: mediolateral oblique

The lesions identified as invasive lobular carcinoma were identified by lesion type, CAD sensitivity, breast parenchyma density, and bilaterality as well as multicentricity (as these carcinomas have a tendency of bilateral and multicentric spread) [21]. The lesion type was identified as a well-formed mass or an ill-defined lesion appearing as architectural distortion. CAD was able to identify a well-formed mass in two mammograms but performed poorly with ill-defined lesions appearing as architectural distortion, which it did not identify in all of the three mammograms the finding was present in. It also missed the multicentric tumor in one out of one mammogram and identified the tumor in the right breast in one of the studies where it missed the lesion in the contralateral breast. It also marked tumors in one view only, that is, the craniocaudal view, in one mammogram and missed the same lesions in the mediolateral oblique projection (Figure 2A-2E). Similar to invasive ductal carcinoma, CAD performed poorly in patients with dense breasts in invasive lobular carcinoma; a dense breast was seen in two out of three mammograms where the pathology was missed in this category of patients. The overall sensitivity of CAD for invasive lobular carcinoma detection was poor and fared at 40% in our study.

The lesions identified as invasive mammary carcinoma were identified by lesion type, CAD detection sensitivity, and breast parenchyma density. Lesion type included four masses only without microcalcification and one mammogram with pleomorphic clusters of microcalcification only. The sensitivity of CAD in the detection of invasive mammary carcinoma was excellent as it was able to identify all four of the masses with this histopathology, as well as one out of one mammogram with pleomorphic clusters of microcalcification only without well-formed mass. Dense breast was present in two out of four mammograms with invasive lobular carcinoma; however, CAD was still able to mark the lesion reliably. The mammogram with pleomorphic clusters of microcalcification only also had asymmetric increased density in the quadrant where this pathology was observed; however, it was marked reliably by CAD.

CAD performance: Normal mammograms

Among the 40 normal mammograms, CAD marked false-positive findings in 20 patients. Hence, the overall false-positive rate was 0.5. Fifteen out of these 20 mammograms had either type C or D parenchyma or type B parenchyma with asymmetric increased density in one of the breast quadrants that the system erroneously identified as a lesion. In the rest of the five mammograms, CAD marked summated normal breast parenchyma as a lesion in only one of the views. The false-positive markings included the mark for mass in 16 of the mammograms (0.4 false-positive marks) and the mark for calcification without mass in the rest of the four mammography images (0.1 false-positive marks) (Table 3).

**Table 3:** False-positive marks identified in normal mammograms in our study

False-positive marking	Number of mammograms marked	False-positive mark
Mass	16	0.4
Calcification without mass	4	0.1

## Discussion

The incorporation of artificial intelligence in radiology for the detection of pathology has led to significant improvement in cancer imaging, in particular where imaging is concerned with repetitive tasks such as screening, tedious tasks such as measurements of lesions, and burdensome work such as detailing the tumor margins [22]. One such software incorporated in mammography is CAD, which, according to literature, has increased breast cancer detection by >20% [23,24]. CAD scans digital mammograms and marks suspicious areas of potential cancer features including masses and microcalcifications. The images are subsequently reviewed by the radiologists who make their own interpretation and then compare the findings with those marked by CAD to come to a conclusion regarding the final impression of the mammogram. Reduced false-negative reporting and increased early detection of breast cancer at an early stage is the intended outcome as the best protection is early detection.

The performance of CAD is better in marking lesions of malignant etiology compared to non-malignant pathology [25]. A review of the literature has shown that indistinct lesions and invasive lobular carcinomas have a greater probability of being missed while reporting mammograms [26,27]. Hence, we conducted this study to identify how useful CAD is in our experience in detecting malignant lesions, irregular mitotic lesions/ill-defined areas of architectural distortion, isolated clusters of cancerous microcalcification or foci of macrocalcification, or combined lesions (mass with calcification).

We saw that CAD had a greater sensitivity in detecting combined lesions (lesions with foci of microcalcification or macrocalfication) and was excellent in identifying and marking clusters of microcalcification, a finding that was similar to the study of Brem et al [18]. The CAD showed better performance for the detection of microcalcification clusters over the detection of lesions. In our experience, the system did not perform well in detecting invasive lobular carcinoma, contrary to Brem et al. [18] and Dromain et al. [28], which showed a superior detection rate for this type of carcinoma. This was especially true when the lesions were ill-defined and subtle, as mammograms of patients with well-circumscribed invasive lobular carcinomas were marked fairly well.

**Figure 3:**
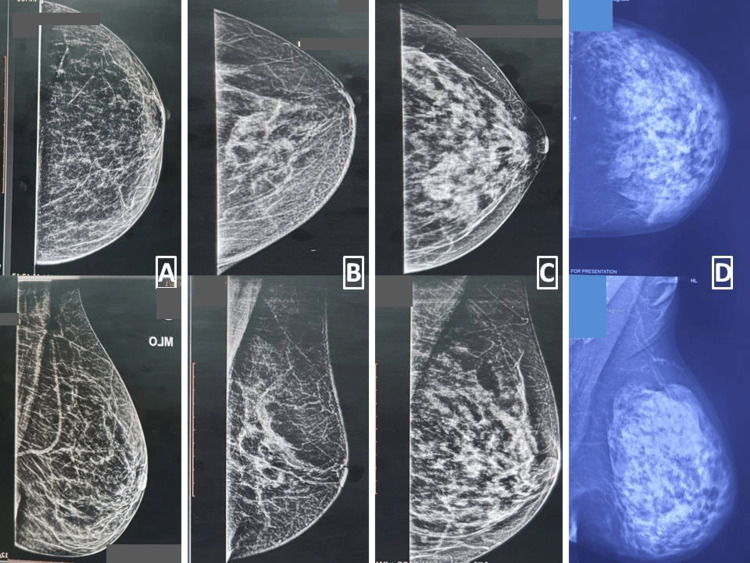
Type A, B, C, and D breast parenchyma in CC and MLO views (CC views above and MLO views below) CC: craniocaudal, MLO: mediolateral oblique

A review of the literature shows contrary results of sensitivity of CAD in dense breasts, with some studies confirming a reduced sensitivity with an increase in density [29,30], while others showing no effect of breast density on lesion detection [31,32]. Areas of asymmetric increased density in any of the quadrants or parenchymal summation artifacts are incorrectly marked as lesions with the use of CAD. Hence, the use of CAD is not recommended in asymmetric areas of increased density and type C or D breast parenchyma. Craniocaudal (CC) and mediolateral oblique (MLO) views of different types of breasts are shown in Figure 3A-3D.

**Figure 4:**
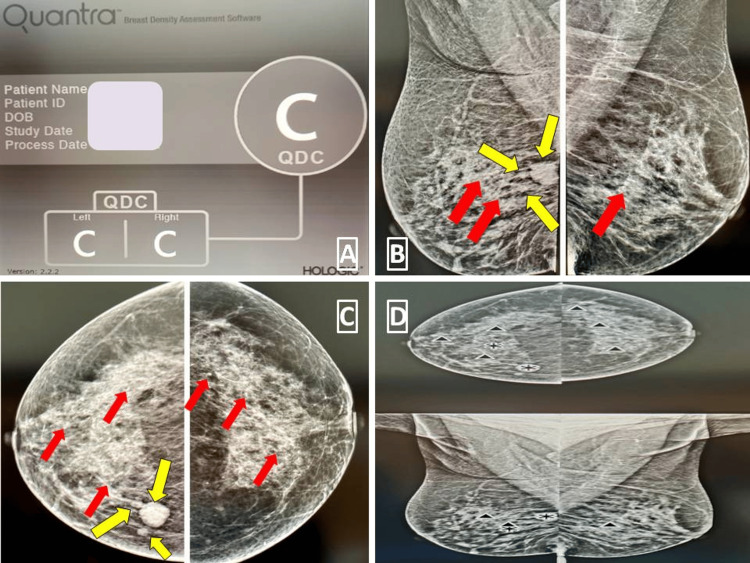
Mammogram images displaying dense breast parenchyma (ACR category C) that may obscure fine details as shown by CAD in Figure 4A At least three small well-circumscribed lesions are observed in the right breast parenchyma in the lower inner quadrant as seen in Figure 4B and Figure 4C, which are lying side by side (best seen in Figure 4C) as indicated by yellow arrows. In addition, scattered foci of macrocalcification are present in both breasts (red arrows). Figure 4D shows that CAD has incorrectly identified an area of parenchymal summation as pathology in the lower inner quadrant of the right breast and marked it as a lesion containing calcification and another area of dense parenchyma as a mass. In addition, although CAD has correctly marked the larger of the three lesions lying side by side, it failed to mark the smaller lesions. Scattered foci of macrocalcification are identified correctly in the left breast. ACR: American College of Radiology, CAD: Computer-Aided Detection

In our experience, increasing the density of the breast had opposite effects on the performance of the system, with an increase in false-positive markings in dense breast parenchyma (Figure 4A-4D). In case of doubtful markings, correlative sonomammography and additional views of mammography are helpful in ruling out suspicious pathology. The sensitivity of CAD increases with increasing lesion size, a finding consistent with previous studies [33]. We found CAD good at detecting pathology in predominant fatty breast parenchyma. CAD was also not reliable in edematous breast parenchyma and erroneously marked coarse parenchymal trabeculae as lesions while missing the true lesion itself. It also missed a retroareolar lesion adherent to the nipple in one of our patients where the lesion size measured up to 1 cm.

Since second reading in mammography is resource-intensive [34], CAD may be a useful addition to assist the reporting radiologist in detecting malignant pathology. However, it cannot entirely be relied on because of its relatively poor performance in ill-defined lesions, subtle carcinomas, dense breasts, and parenchymal summation artifacts. The reporting radiologist must be aware of the potential strengths and pitfalls for better utilization of the system and to avoid potential hazards of missed lesions or overreporting a carcinoma. Human intelligence has hence beaten CAD for the diagnosis of breast malignancy in mammograms.

The potential limitations of our study were a small sample size, an inability to collect an equal number of patients for all tumor types, an inability to collect an equal number of data for ill-defined lesions versus well-circumscribed lesions, and it being a single-institution study. Future studies with the inclusion of a larger number of patients, multicenter trials, and equity in lesion type and tumor type will be helpful.

## Conclusions

Newer advances such as AI software are becoming increasingly popular for problem-solving and enhancing lesion detection, as early detection and treatment have better prognosis and survival. In our experience, the use of one such software, CAD, has excellent performance in detecting suspicious clusters of microcalcification as well as patients with ACR type A and type B breast parenchyma. However, it does not fare well in patients with dense breasts (ACR type C and D), asymmetric increased breast densities, edematous breast parenchyma, irregular/ill-defined lesions of invasive lobular carcinoma, and retroareolar lesions. Hence, although CAD may be a useful supplementary tool to assist the reporting radiologist in detecting malignant pathology, it cannot entirely be relied on, and it is essential for reporting radiologists to recognize its strengths and weaknesses to avoid false-positive or false-negative reporting. This research adds a cautionary note to existing literature that largely promotes the efficacy of CAD systems in improving cancer detection rates.
